# Prognosis of clear cell renal cell carcinoma patients stratified by age: A research relied on SEER database

**DOI:** 10.3389/fonc.2022.975779

**Published:** 2022-10-12

**Authors:** Zhouning Liao, Dang Wang, Ning Song, Yang Xu, Heming Ge, Zhangzhe Peng

**Affiliations:** ^1^ Department of Nephrology, Xiangya Hospital, Central South University, Changsha, China; ^2^ Department of General Surgery, Xiangya Hospital, Central South University, Changsha, China; ^3^ Division of Translational Immunology, III, Department of Medicine, University Medical Center Hamburg-Eppendorf, Hamburg, Germany; ^4^ Department of General, Visceral and Thoracic Surgery, University Medical Center Hamburg-Eppendorf, Hamburg, Germany

**Keywords:** Clear cell renal cell carcinoma, seer, age, prognosis, all-cause mortality, tumor-specific mortality

## Abstract

**Objective:**

Clear cell renal cell carcinoma may affect patients of any age. To date, there are only a limited number of large data studies on renal clear cell carcinoma in different age groups. This study assessed CCRCC risk factors in different age groups using the Surveillance Epidemiology and End Results (SEER) database.

**Methods:**

We selected 58372 cases from the SEER database. These patients were divided into seven different age groups. Cox regression models were used to find independent risk factors for the survival of CCRCC patients. Based on independent risk factors, a nomogram was drawn with R software. Kaplan-Meier method for survival analysis and X-tile software were used to find the optimal age group for diagnosis.

**Results:**

Univariate analysis revealed that patients’ age, sex, race, marital status, grade, TNM (tumor, node, metastasis) stage, surgery, WHO/ISUP grade were correlated with survival (P<0.01). Age was an independent risk factor for survival in patients with CCRCC according to multivariate Cox regression analysis (p<0.01). All-cause mortality and tumor-specific mortality increased according to the increasing age of the patients. The optimal cut-off values for age were defined as 58 and 76 years and 51 and 76 years, respectively, according to overall survival (OS) and cause-specific survival (CSS).

**Conclusion:**

There is a negative correlation between age and survival of CCRCC patients. The difference in prognosis of patients in different age groups has important implications for clinical treatment. Therefore, the diagnosis and treatment plan should be based on more detailed age grouping, which is more beneficial to improving the prognosis and survival of patients.

## Introduction

Renal cell carcinoma (RCC) affects >430,000 people worldwide every year (1), the incidence is about twice as high in men as in women (2).RCC includes chromophobe renal cell carcinoma (CHRCC), papillary renal cell carcinoma (PRCC), and clear cell renal cell carcinoma (CCRCC), which are three main histological subtypes with distinct molecular and genetic characteristics (3). Among them, CCRCC is the most prevalent histological subtype (approximately 80%).

Age is an indicator used to assess prognosis in many solid cancers, especially in CCRCC, and is a significant risk factor (4, 5). CCRCC may affect patients of any age, but it is more likely to affect people aged 60-70 years, with younger people rarely being affected. Meanwhile, the prognosis of CCRCC in older patients is poor, and patients are usually diagnosed at an advanced stage, which may be related to the insidious nature of CCRCC symptoms and low level of diagnosis (6–8). There have been many studies on risk factors for the prognosis of patients with CCRCC, which have revolutionized the treatment options for CCRCC. Population based data suggest that the incidence of CCRCC varies by age group. In addition, a comparison of the vascular features of patients aged <65 and >65 years has shown that age can affect the molecular features and vascular structure of CCRCC (9).

There have been several achievements regarding the role of age in patients with CCRCC. However, data on CCRCC in different age groups are limited. This study used the constructed SEER database to assess the risk factors for CCRCC in various age groups.

## Materials and methods

Data Sources together with Selection Criteria CCRCC clinical data were acquired from the SEER database through employing the SEER Stat 8.3.9 software (https://seer.cancer.gov/data/). SEER is the authoritative source of the cancer statistics in the U.S., with its database covering socioeconomic status, population statistics, incidence rate and survival rate among the cancer patients. The patient data included: The patient ID, marital status, sex, grade, American Joint Committee on Cancer (AJCC) TNM stage, surgery, WHO/ISUP grade, survival (months). The database used the 7th (2010–2015) and 6th (2004–2015) TNM staging systems from 2004 to 2015, so we converted the 6th edition to the 8th edition based on CS Extension and CS Lymph Nodes.

Inclusion criteria: (1) Histologically diagnosed with CCRCC. (2) the year at time of diagnosis was 2004-2015.

Exclusion criteria: (1) Non-pathological diagnosis and patient was not CCRCC; (2) T stage=T0, Tis, Tx, NA or N stage= Nx, NA or M stage=Mx, NA; (3) patient without surgery or unknown.

### Variables and results

The variables included marital status, sex, grade, T stage, N stage, M stage, Surgery, WHO/ISUP grade, Survival (months).These patients were classified as seven groups in accordance with age, that is, 0-30, 30-39, 40-49, 50-59, 60-69, 70-79, and above 80. Subgroup analysis was conducted by marital status(unmarried vs. married vs. unknown), sex(male vs. female), grade(grade I vs. grade II vs. grade III vs. grade IV), T stage(T1 vs. T2 vs. T3 vs. T4), N stage(N0 vs. N1), M stage(M0 vs. M1), surgery(no surgery vs. local tumor excision vs. partial nephrectomy vs. complete nephrectomy), WHO/ISUP grade(low grade vs. high grade vs. unknown). The survival analysis was implemented, containing cancer special survival together with overall survival.

### Statistical method

With an aim of comparing the baseline features of patients in various age groups, Fisher test and Chi-square test were applied. In the univariate analysis, Kaplan Meier approach was utilized to explore the survival rate under each factor of risk. Chi-square test was employed for the comparison between groups. In univariate analysis, univariate Cox regression model was applied to assess the prognostic risk factors. In the multivariate analysis, Cox regression model was exploited to analyze the independent risk factors of survival in patients with CCRCC. R software (version 4.1.3) and SPSS 26.0 software were conducted for complete analysis. Eventually, X-tile software (version 3.6.1) was applied to classify the patients as three subgroups, namely, low risk, medium risk and high risk. No ethical statement consent was required for this study, as the SEER data were publicly available and analyzed anonymously. All authors signed the research agreement form and had access to the SEER database.

## Results

### Baseline characteristics of the patients

**Figure 1:**
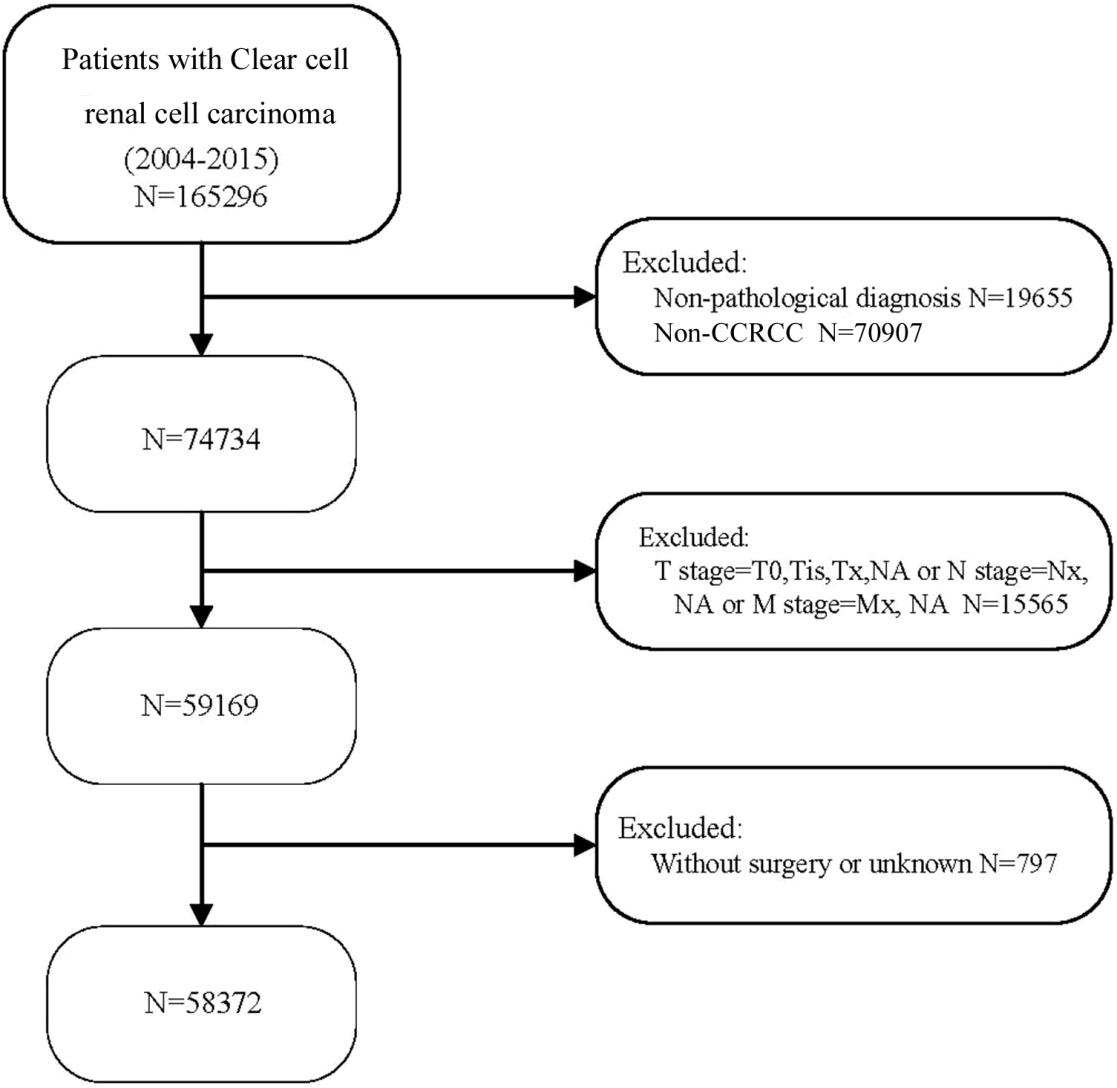
Flow chart for selecting patients with CCRCC. 2004-2015 CCRCC patients were selected in detail from the SEER database.

58,372 eligible patients with CCRCC were acquired (**Figure 1**). 412 cases, aged between 0 and 29 years; 2348 patients aged 30 to 39, 7572 patients aged between 40 and 49, and 14729 patients aged 50 to 59, 17575 patients aged 60 to 69, 11908 patients aged between 70 and 79 together with 3828 patients aged 80 and over. Compared with patients aged below 30 years, patients over 80 years of age had a higher rate of node-positive tumors (2.9% vs. 2.4%; P<0.001), gradeIII tumors (25.1% vs 16.1%; P<0.001) and gradeIV tumors (5.2% vs 1.9%; P<0.001). Meanwhile, patients aged 80 years and above less frequently harbored T1(86.4% vs. 64%), and more frequently harbored T2(8.9% vs. 9.1%), and T3 (3.8% vs. 25.1%;all P<0.001). In contrast to patients under 60 years of age, patients over 80 years of age are more frequently diagnosed at M1 (7.5% vs 0.9%). Notably, among the seven different age groups, patients aged 60-69 years had the highest percentage of node-positive tumor (3.5%), gradeIII tumors (25.3%), T4(1.1%) and M1(9.0%). The highest rates of grade IV tumors (5.9%) and T4 (1.1%) were found in patients aged 50-59 years. Patients’ data include gender, marital status, race, grade, TNM stage, surgery and WHO/ISUP grade. See (**Table 1**) for a summary.

**Table 1:** Patient demographics and clinical characteristics.

Variance	0-30yrs (n=412)	30-39yrs (n=2348)	40-49yrs (n=7572)	50-59yrs (n=14729)	60-69yrs (n=17575)	70-79yrs (n=11908)	≥80yrs (n=3828)	Total (n=58372)	p values
	Number (%)	Number (%)	Number (%)	Number (%)	Number (%)	Number (%)	Number (%)	Number (%)	
**sex**									<0.001
male	214 (51.9)	1423 (60.6)	4835 (63.8)	9491 (64.4)	11074 (63)	7083 (59.4)	2165 (56.5)	36285 (62.1)	
female	198 (48)	925 (39.3)	2737 (36.1)	5238 (35.5)	6501 (36.9)	4825 (40.5)	1663 (43.4)	22087 (37.8)	
**Race**									<0.001
white	`	1964 (83.6)	6341 (83.7)	12355 (83.8)	14785 (84.1)	10238 (85.9)	3352 (87.5)	49374 (84.5)	
black	36 (8.7)	158 (6.7)	582 (7.6)	1213 (8.2)	1388 (7.8)	753 (6.3)	173 (4.5)	4303 (7.3)	
others	34 (8.2)	189 (8.0)	584 (7.7)	1084 (7.3)	1312 (7.4)	873 (7.3)	297 (7.7)	4373 (7.4)	
unkown	3 (0.7)	37 (1.5)	64 (0.8)	77 (0.5)	90 (0.5)	44 (0.3)	6 (0.1)	322 (0.5)	
**Marital status**									<0.001
unmarried	264 (64)	911 (38.7)	2608 (34.4)	4572 (31)	5125 (29.1)	3804 (31.9)	1683 (43.9)	18967 (32.4)	
married	121 (29.3)	1321 (56.2)	4573 (60.3)	9479 (64.3)	11559 (65.7)	7609 (63.8)	1982 (51.7)	36644 (62.7)	
unknown	27 (6.5)	116 (4.9)	391 (5.1)	678 (4.6)	891 (5)	495 (4.1)	163 (4.2)	2761 (4.7)	
**Grade**									<0.001
gradeI	69 (16.7)	349 (14.8)	1088 (13.3)	1622 (11)	1838 (10.4)	1370 (11.5)	418 (10.9)	6674 (11.4)	
gradeII	230 (55.8)	1319 (56.1)	3866 (51)	6981 (47.3)	8348 (47.4)	5547 (46.5)	1658 (43.4)	27949 (47.8)	
gradeIII	68 (16.5)	398 (16.9)	1609 (21.2)	3703 (25.1)	4456 (25.3)	2909 (24.4)	964 (25.1)	14107 (24.1)	
gradeIV	8 (1.9)	69 (2.9)	356 (4.7)	870 (5.9)	1016 (5.7)	644 (5.4)	202 (5.2)	3165 (5.4)	
unknown	37 (8.9)	213 (9.0)	733 (9.6)	1553 (10.5)	1917 (10.9)	1438 (12)	586 (15.3)	6477 (11.0)	
**T stage**									<0.001
T1	357 (86.6)	1944 (82.7)	5530 (73)	9942 (67.4)	11741 (66.8)	8005 (67.2)	2452 (64)	39971 (68.4)	
T2	37 (8.9)	210 (8.9)	876 (11.5)	1699 (11.5)	1738 (9.8)	1073 (9.0)	352 (9.1)	5985 (10.2)	
T3	16 (3.8)	185 (7.8)	1122 (14.8)	2916 (19.7)	3889 (22.1)	2710 (22.7)	962 (25.1)	11800 (20.2)	
T4	2 (0.4)	9 (0.3)	44 (0.5)	172 (1.1)	207 (1.1)	120 (1)	62 (1.6)	616 (1.0)	
**N stage**									<0.001
N0	402 (97.5)	2312 (98.4)	7369 (97.3)	14235 (96.6)	16950 (96.4)	11599 (97.4)	3715 (97)	56582 (96.9)	
N1	10 (2.4)	36 (1.5)	203 (2.6)	494 (3.3)	625 (3.5)	309 (2.5)	113 (2.9)	1790 (3.0)	
**M stage**									<0.001
M0	408 (99)	2289 (97.4)	7105 (93.8)	13419 (91.1)	15991 (90.9)	11029 (92.6)	3540 (92.4)	53781 (92.1)	
M1	4 (0.9)	59 (2.5)	467 (6.1)	1310 (8.8)	1584 (9.0)	879 (7.3)	288 (7.5)	4591 (7.8)	
**Surgery**									<0.001
No surgery	8 (1.9)	32 (1.3)	152 (2.0)	482 (3.2)	784 (4.4)	670 (5.6)	445 (11.6)	2573 (4.4)	
Local tumor excision	11 (2.6)	57 (2.4)	142 (1.8)	383 (2.6)	684 (3.8)	743 (6.2)	360 (9.4)	2380 (4.0)	
Partial nephrectomy	232 (56.3)	1169 (49.7)	2965 (39.1)	4690 (31.8)	5259 (29.9)	2937 (24.6)	548 (14.3)	17800 (30.4)	
Complete nephrectomy	161 (39)	1090 (46.4)	4313 (56.9)	9176 (62.2)	10848 (61.7)	7558 (63.4)	2475 (64.6)	35619 (61.0)	
WHO/ISUP Grade*									<0.001
Low grade	2 (0.4)	21 (0.8)	171 (2.2)	476 (3.2)	625 (3.5)	439 (3.6)	167 (4.3)	1901 (3.2)	
High grade	3 (0.7)	27 (1.1)	125 (1.6)	344 (2.3)	476 (2.7)	323 (2.7)	101 (2.6)	1399 (2.3)	
unknown	407 (98.7)	2300 (97.9)	7276 (96)	13909 (94.4)	16474 (93.7)	11146 (93.6)	3560 (92.9)	55072 (94.3)	

*WHO/ISUP indicates World Health organization/International Society of Urological Pathology.

### Influence of age on cancer special survival and overall survival

**Figure 2:**
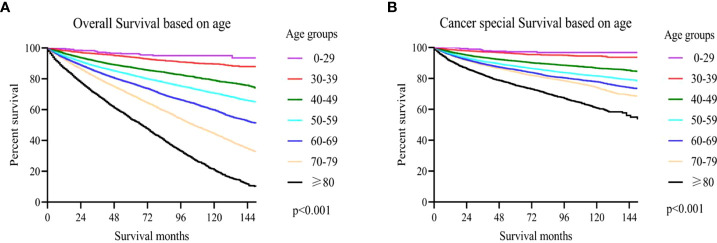
**(A)** The overall survival (OS) based on age upon diagnosis; **(B)** cancer-specific survival (CSS) based on age upon diagnosis.

In accordance with Kaplan-Meier curves, both cancer special survival (CSS) and overall survival (OS) were revealed to decrease with age (P<0.001; **Figure 2**). In univariate analysis, it was found that in addition to age, gender, marital status, race, grade, TNM stage, surgery and WHO/ISUP grade were also correlated with survival (OS and CSS). Analysis of these variables using Cox regression and Kaplan-Meier curves revealed that age, gender, marital status, race, grade, TNM stage, surgery and WHO/ISUP grade were also the independent risk factors of survival (P<0.01; **Tables 2**, **3**; **Figures S1**, **S2**).

**Table 2:** Uni- and Multivariate Cox Regression Analysis of OS (n=58372).

Variance	Univariate analysis			Multivariate analysis		
	P value	HR*	95% CI*	P value	HR*	95% CI*
**Sex**	<0.001	1.193	1.156-1.231			
male				Reference		
female				<0.001	1.18	1.142-1.219
**Race**	<0.001	0.926	0.902-0.951	<0.001		
white				Reference		
black				<0.001	1.131	1.069-1.198
others				<0.001	0.892	0.839-0.947
unkown				<0.001	0.223	0.132-0.377
**Age**	<0.001	1.524	1.505-1.544	<0.001		
0-30yrs				Reference		
30-39yrs				<0.005	2.006	1.235-3.258
40-49yrs				<0.001	3.551	2.229-5.657
50-59yrs				<0.001	4.613	2.901-7.333
60-69yrs				<0.001	6.575	4.138-10.449
70-79yrs				<0.001	10.222	6.432-16.243
≥80yrs				<0.001	15.777	9.918-25.095
**Marital status**	<0.001	0.779	0.758-0.801	<0.001		
unmarried				Reference		
married				<0.001	0.752	0.728-0.777
unknown				<0.001	0.723	0.665-0.785
**Grade**	<0.001	1.309	1.309-1.341	<0.001		
gradeI				Reference		
gradeII				0.486	1.02	0.965-1.079
gradeIII				<0.001	1.31	1.234-1.390
gradeIV				<0.001	2.064	1.919-2.221
unknown				<0.001	1.167	1.092-1.248
**T stage**	<0.001	1.745	1.717-1.773	<0.001		
T1				Reference		
T2				<0.001	1.359	1.294-1.427
T3				<0.001	1.605	1.540-1.672
T4				<0.001	1.945	1.765-2.143
**N stage**	<0.001	7.490	7.094-7.909			
N0				Reference		
N1				<0.001	1.922	1.808-2.044
**M stage**						
M0				Reference		
M1				<0.001	3.838	3.663-4.021
**Surgery**	<0.001	8.266	7.962-8.580	<0.001		
No surgery				Reference		
Local tumor excision				<0.001	0.406	0.370-0.446
Partial nephrectomy				<0.001	0.23	0.214-0.248
Complete nephrectomy				<0.001	0.352	0.331-0.374
WHO/ISUP Grade*	<0.001	0.793	0.777-0.809	<0.001		
Low grade				Reference		
High grade				0.035	0.782	0.694-0.881
unknown				<0.001	0.919	0.850-0.994

*WHO/ISUP indicates World Health organization/International Society of Urological Pathology; HR, hazard ratio; CI, confidence interval.

**Table 3:** Uni- and Multivariate Cox Regression Analysis of CSS (n=58372).

Variance	Univariate analysis			Multivariate analysis		
	P value	HR*	95% CI*	P value	HR*	95% CI*
**Sex**	<0.001	1.286	1.230-1.344			
male				Reference		
female				<0.005	1.068	1.020-1.119
**Race**	<0.001	0.929	0.896-0.964	<0.005		
white				Reference		
black				0.491	1.030	0.946-1.121
others				0.172	0.945	0.871-1.025
unkown				<0.001	0.306	0.159-0.588
**Age**	<0.001	1.314	1.291-1.337	<0.001		
0-30yrs				Reference		
30-39yrs				0.013	1.654	1.088-3.083
40-49yrs				<0.001	2.677	1.476-4.858
50-59yrs				<0.001	3.106	1.716-5.622
60-69yrs				<0.001	3.770	2.084-6.820
70-79yrs				<0.001	4.831	2.669-8.743
≥80yrs				<0.001	6.966	3.842-12.630
**Marital status**	<0.001	0.846	0.814-0.880	<0.001		
unmarried				Reference		
married				<0.001	0.840	0.803-0.880
unknown				<0.001	0.719	0.636-0.813
**Grade**	<0.001	1.583	1.558-1.609	<0.001		
gradeI				Reference		
gradeII				<0.001	1.188	1.073-1.316
gradeIII				<0.001	2.005	1.808-2.223
gradeIV				<0.001	3.371	3.010-3.775
unknown				<0.001	1.633	1.461-1.826
**T stage**	<0.001	2.568	2.513-2.625	<0.001		
T1				Reference		
T2				<0.001	2.275	2.128-2.431
T3				<0.001	2.769	2.612-2.935
T4				<0.001	3.273	2.939-3.645
**N stage**	<0.001	12.462	11.738-13.231			
N0				Reference		
N1				<0.001	1.830	1.712-1.957
**M stage**	<0.001	16.874	16.141-17.642			
M0				Reference		
M1				<0.001	5.324	5.036-5.629
**Surgery**	<0.001	0.739	0.720-0.759	<0.001		
No surgery				Reference		
Local tumor excision				<0.001	0.296	0.251-0.348
Partial nephrectomy				<0.001	0.157	0.141-0.175
Complete nephrectomy				<0.001	0.291	0.270-0.315
WHO/ISUP Grade*	<0.001	0.584	0.561-0.609	<0.001		
Low grade				Reference		
High grade				<0.001	0.770	0.671-0.885
unknown				0.562	0.973	0.888-1.067

* WHO/ISUP indicates World Health organization/International Society of Urological Pathology; HR, hazard ratio; CI, confidence interval.

### Nomogram for forecasting OS and CSS in CCRCC patients

**Figure 3:**
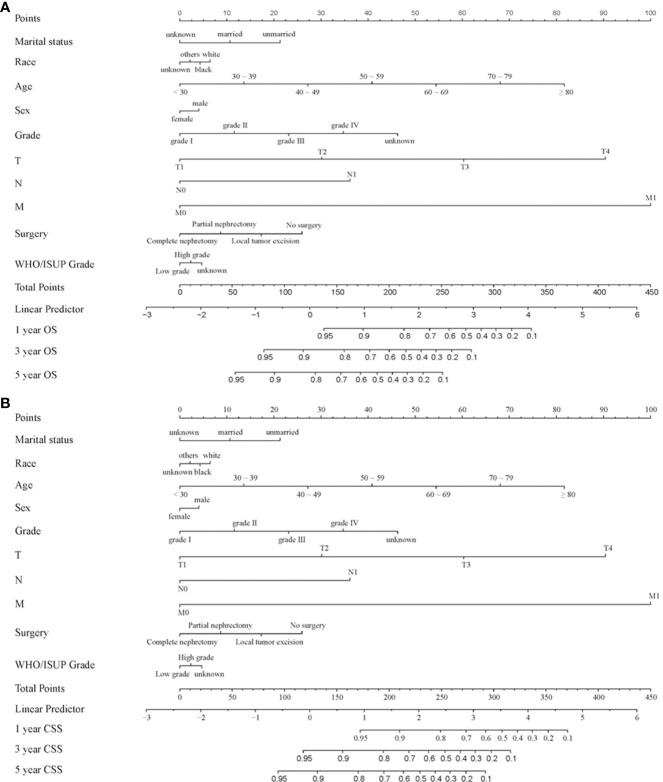
Nomogram for forecasting OS and CSS in CCRCC patients. **(A)** Nomogram forecasting 1-year, 3-year, and 5-year OS in CCRCC patients; **(B)** Nomogram forecasting 1-year, 3-year, and 5-year CSS in CCRCC patients.

Nomograms are widely applied in cancer prognosis, principally owing to they can simplify the model of statistical prediction into a single numerical estimation of the event probability (recurrence or death), which is tailored to the patients’ condition. In the training set, ten variables form a nomogram. The specific phases of utilizing the nomogram are as follows: based on the classification (for instance, marital status is classified as unmarried, married and unknown) of each prognostic variable (age, gender, marital status, race, grade, TNM stage, WHO/ISUP grade and surgery), a vertical line was drawn at the top of the nomogram on the horizontal axis labeled “points”. Each prognostic variable was assigned a score at the site where vertical line crosses the “point” axis. The total score was the sum of the scores of the ten variables. Drawn a vertical line on the horizontal axis labeled “Total Score” with the 1-, 3- and 5-year OS as the axis. The intersection of the 1-year OS axis with the vertical line represents the 1-year OS rate (**Figure 3**).

### X-tile analysis determines the optimal cut-off value for age

**Figure 4:**
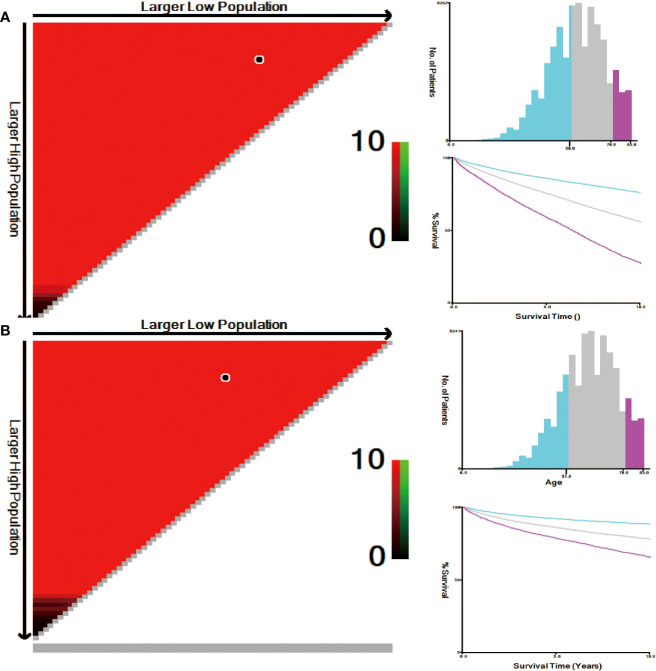
**(A)** Optimal cut-off point for OS defined with X-tile software; **(B)** Optimal cut-off point for CSS defined with X-tile software.

The correlation between patient age and mortality was explored by using X-tile software. The plots were generated through randomly classifying the age as three groups: low, medium and high. The best dividing point was determined ***via*** black/white circles on the χ ^2^ axis. It was concluded that the optimal cut-off values for age based on overall survival (OS) were determined to be 58 and 76 years. Kaplan-Meier survival curves were formulated according to the age subgroups of OS (**Figure 4A**); Simultaneously, the best age cut-off depending on cause-specific survival (CSS) was 51 and 76 years and the survival curves were drawn for these age subgroups of CSS using the Kaplan Meier method (**Figure 4B**).

## Discussion

Despite many studies on CCRCC, limited studies have reported novel findings on age as a prognostic factor for CCRCC and many studies have reported widely divergent findings. To our knowledge, no studies have independently analyzed the prognostic differences between different age groups of patients with CCRCC using a large database. Various studies have shown that children and adults show different features and outcomes of CCRCC. Age is a significant prognostic factor in patients with CCRCC (10–12). The current studies have focused on children, adults, or a particular age point and ignored the differences between age groups (6, 10, 11, 13). Several studies have shown that younger patients with RCC have better pathological characteristics and histological subtypes as well as significantly lower disease-specific mortality than older patients (13–16). Notably, in the treatment decision-making of patients with CCRCC, older patients should be provided with better treatment options and more attention. In contrast to young people, older patients have worse treatment results, lower survival rates, and a greater deterioration risk. This is probably associated with the poor physical activity or underlying diseases in older patients (6). Many studies have found differences between young and older people, but no studies have reported differences between age groups and their impact on the prognosis of patients with CCRCC (15, 17, 18). Several studies comparing mortality rates in older and younger cohorts have found that mortality rates were significantly higher in older adults than in younger adults. Therefore, it has been hypothesized that mortality in patients with CCRCC increases with age (17, 19). This hypothesis is supported by the outcomes of our study, revealing an evident increase in both tumor-specific and all-cause mortality with age in seven different age subgroups of patients with CCRCC, analyzed using univariate and multivariate analyses. Therefore, we recommend individualized treatment for patients with CCRCC to achieve better survival.

The present study included 58,372 patients with CCRCC from the SEER database. According to our age grouping, we found that the TNM stage gradually increased with age. However, patients aged 60-69 years had the highest percentage of node-positive tumor, gradeIII tumors, T4 and M1. The highest rates of grade IV tumors and T4 were found in patients aged 50-59 years. Moreover, 51 and 76 years were used as the cutoff age according to cancer-specific survival rates. The results of the survival analysis suggested that younger age was an independent predictor of CCRCC.

Several studies have suggested a higher stage, smaller size and lower nuclear grade in older patients aged over 50 years with RCC (11). A cutoff age of 40 years to categorize young and older patients with CCRCC. Taccoen et al. observed that age <40 years is a CCRCC-independent prognostic factor, and the prognosis is better (12). Aziz et al. discovered a significantly lower rate of all etiologies, morbidity, and mortality in young patients aged <40 years with RCC (20). Kang et al. also discovered that young age was related to good pathological features, although it was not an independent prognostic factor for survival in patients with surgically treated RCC. Nevertheless, the outcomes of Kaplan–Meier analysis indicated that the younger group aged <40 years had significantly better CCS rates than the middle-aged group aged 40–60 years and older group aged ≥60 years (13). Kim et al. found a more favorable histological subtype for young patients with RCC aged 20–39 years than for those aged 40–79 years (17). Komai et al. have reported that 45 years is the best critical cutoff age to categorize patients into the older and younger groups. Although younger patients with RCC have relapse-free survival rates similar to those of older patients, they have higher CSS rates (15). Jung et al. reported that young age was an independent predictor of prognosis according to multivariate analysis. Moreover, 55 years is a critical cutoff age for distinguishing between older and young patients with CCRCC (11). In this study, we evaluated all possible ages using X-tile plots and selected 51 and 76 years as the cutoff age based on cancer-specific survival. The younger group had better overall survival and CSS than the older group.

However, our study has some limitations. First, in the SEER database, studies utilizing population databases have inherent limitations due to heterogeneity in clinical practice at the participating centers. Second, important treatment information is missing from the SEER database, such as the use of systemic therapy, chemotherapy regimens, metastasectomy, dose of radiation therapy, completeness of surgery, or any other information related to treatment intensity. In addition, information on socioeconomic status and other clinical variables is lacking. The major known risk factors for CCRCC are hypertension, obesity, and smoking (21–26). Nonetheless, owing to the lack of associated data in the renal cancer database, we could not analyze these factors. Third, there may be selection bias due to the retrospective nature of this analysis. Therefore, a prospective randomized trial should be designed to test our study hypothesis and to validate or refute any of the above explanations for the observed relationship between age and prognosis in patients with CCRCC. Despite these limitations, our study was a population-based study in which a large number of patients with CCRCC were included and the results were convincing.

## Conclusion

Negative correlation between age and survival of CCRCC patients was found through SEER database. In other words, the older the patient was, the lower the survival rate. Discovering differences in the patients’ prognosis with CCRCC in various age groups has important implications for clinical treatment. Therefore, individualized treatment of patients with CCRCC is imperative. It is unreasonable to distinguish the clear cell renal cell carcinoma only in accordance with the situation of the children, adults and elderly, the diagnosis and treatment plan should be based on more detailed age grouping, which is more beneficial to improving the prognosis and survival rate of the patients.

## Data availability statement

The datasets presented in this study can be found in online repositories. The names of the repository/repositories and accession number(s) can be found below: www.seer.cancer.gov.

## Author contributions

ZL, HG and ZP designed the study. DW, NS, and YX contributed to data selection and assembly. ZL, HG, and ZP analyzed data. ZL and HG were involved in drafting the manuscript. ZP critically revised the manuscript. All authors contributed to the article and approved the submitted version.

## Conflict of Interest

The authors declare that the research was conducted in the absence of any commercial or financial relationships that could be construed as a potential conflict of interest.

## Publisher's note

All claims expressed in this article are solely those of the authors and do not necessarily represent those of their affiliated organizations, or those of the publisher, the editors and the reviewers. Any product that may be evaluated in this article, or claim that may be made by its manufacturer, is not guaranteed or endorsed by the publisher.

## Supplementary material

Supplementary Figure 1 Kaplan-Meier curves for OS according to age for **(A)** grade; **(B)** T stage; **(C)** N stage; **(D) **M stage; **(E)** surgery.

Supplementary Figure 2 Kaplan-Meier curves for CSS according to age for **(A)** grade; **(B)** T stage; **(C)** N stage; **(D) **M stage; **(E)** surgery.

## Abbreviations

CCRCC: Clear cell renal cell carcinoma; PRCC: papillary renal cell carcinoma; CHRCC: chromophobe renal cell carcinoma; KC: kidney cancer; RCC: renal cell cancer; TCC: transitional cell cancer; SEER: Surveillance, Epidemiology, and End Result; OS: overall survival; CSS: cancer special survival.
