# Supplementary figures and images for: Prognosis of clear cell renal cell carcinoma patients stratified by age: A research relied on SEER database
Source: Front Oncol. 2022 Oct 12;12:975779. doi: 10.3389/fonc.2022.975779 (PMC9597499; doi:10.3389/fonc.2022.975779)

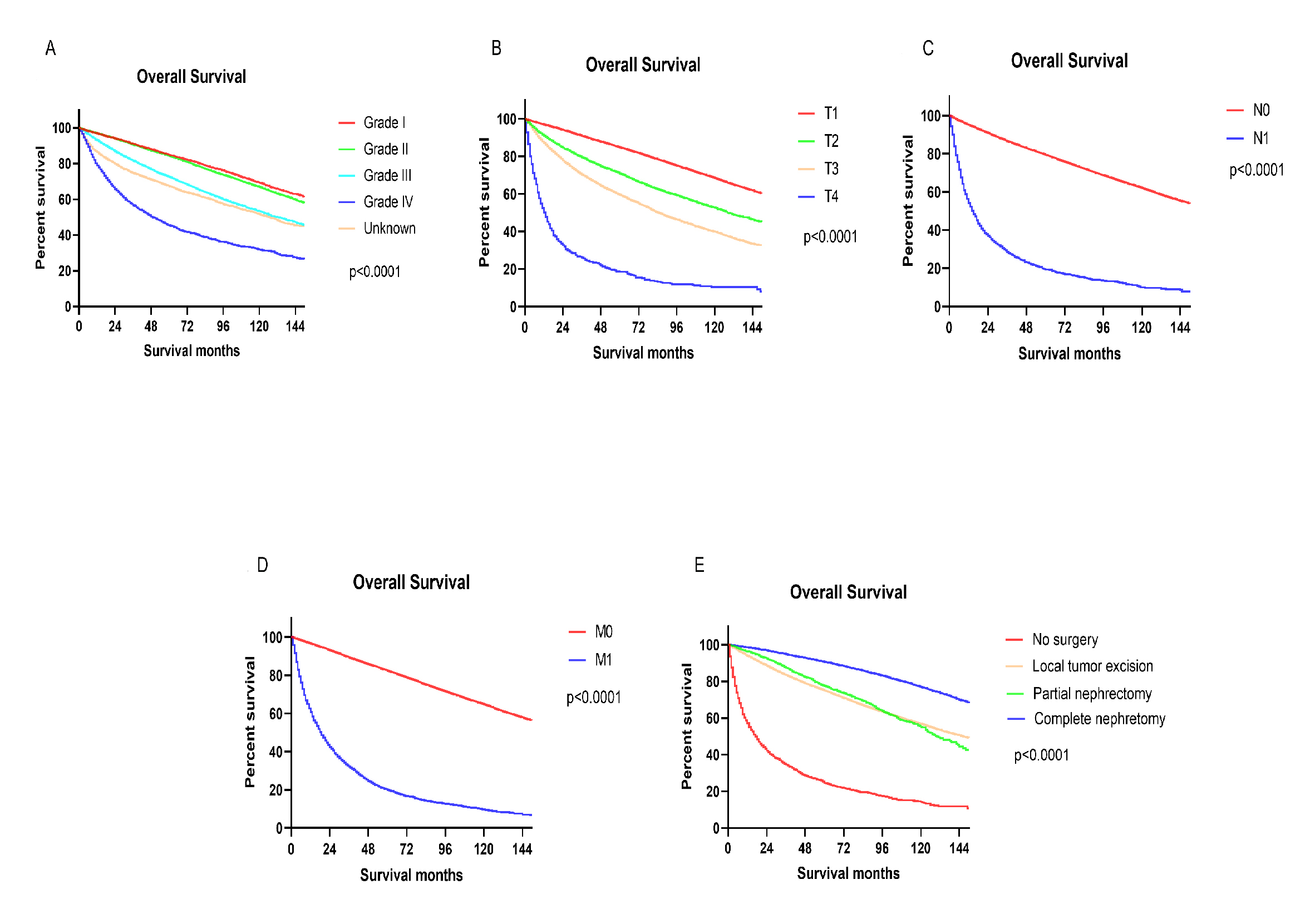

Supplement: Supplementary Figure 1 — Kaplan-Meier curves for OS according to age for (A) grade; (B) T stage; (C) N stage; (D) M stage; (E) surgery. [file Image_1.jpeg]

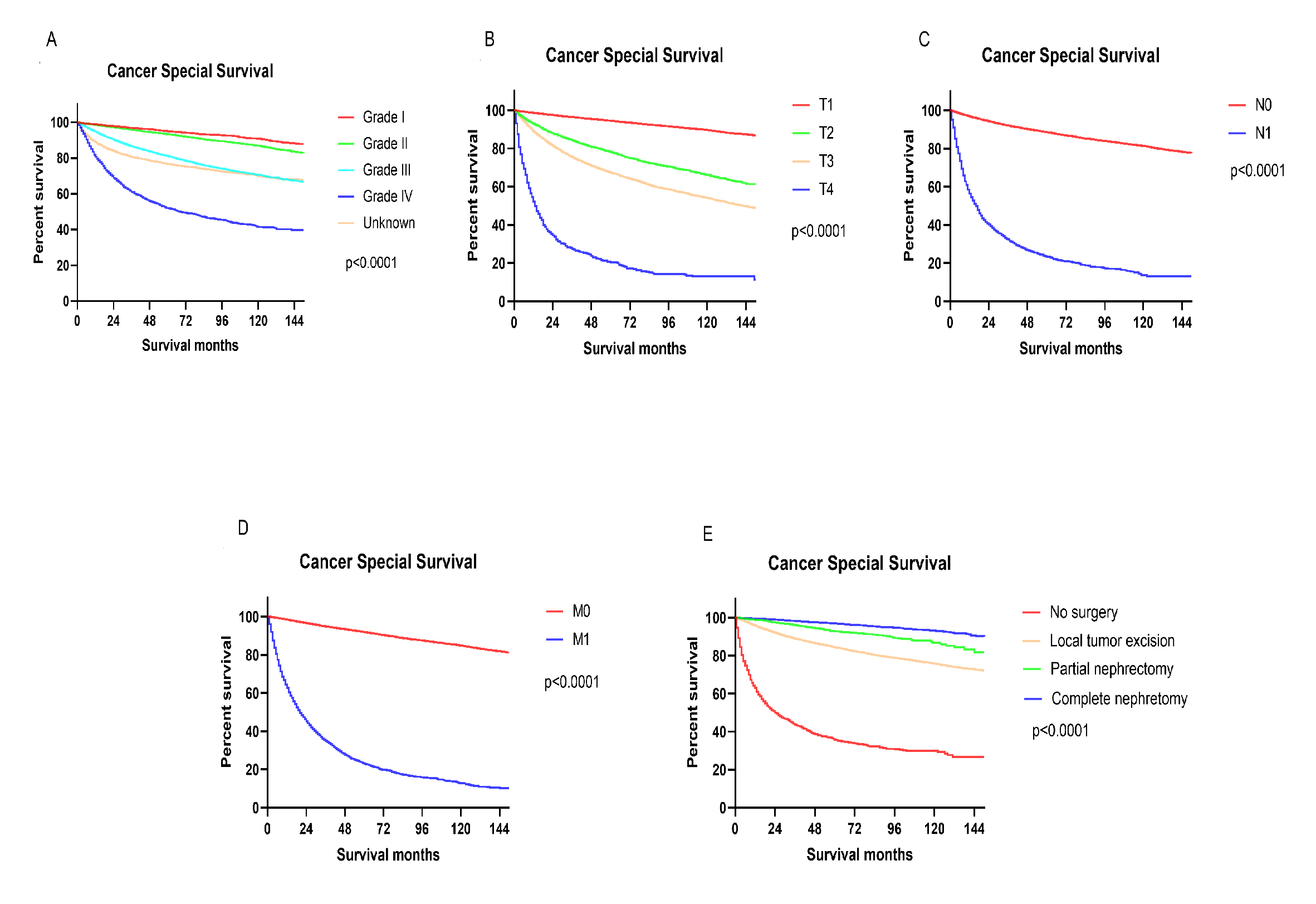

Supplement: Supplementary Figure 2 — Kaplan-Meier curves for CSS according to age for (A) grade; (B) T stage; (C) N stage; (D) M stage; (E) surgery. [file Image_2.jpeg]
